# The first study on the occurrence of bovine herpesviruses in the wild fauna of the Moscow region, Russia

**DOI:** 10.14202/vetworld.2022.2052-2058

**Published:** 2022-08-24

**Authors:** Svetlana P. Yatsentyuk, Alexander V. Pchelnikov, Elizaveta R. Safina, Maria S. Krasnikova

**Affiliations:** Department of Biotechnology, Russian State Center for Animal Feed and Drug Standardization and Quality, Zvenigorodskoe Highway, Moscow, Russia

**Keywords:** antibody, bovine herpesvirus-1, bovine herpesviruses, moose, polymerase chain reaction, roe deer

## Abstract

**Background and Aim:**

Some pathogens that cause infections in cattle are found in wild artiodactyls. Their prevalence, possible impact on the population of free-living animals, and the spread of infectious pathology in livestock have yet to be studied. We investigated the occurrence of bovine herpesviruses (BoHV-1, BoHV-4, and BoHV-6) among wild moose and roe deer in 8 areas of the Moscow region in the Russian Federation.

**Materials and Methods:**

One hundred and one tissue samples and nasal swabs of 24 moose and seven roe deer were studied using a real-time polymerase chain reaction (PCR) for BoHV-1 DNA and conventional PCR for BoHV-4 and BoHV-6 DNA. A virus neutralization test (VNT) was used to detect antibodies to BoHV-1 in 19 serum samples. The final antibody titer was calculated with the Spearman-Kärber method.

**Results:**

BoHV-4 and BoHV-6 DNA were not detected in all studied samples of 31 animals. BoHV-1 DNA was detected using a real-time PCR in nasal swabs from 2 adult roe deer. For BoHV-1, only 9/19 tested serum samples reacted positive in VNT with the titer range from 0.67 ± 0.19 to 3.75 ± 0.10 log2. Antibodies were detected in all age groups, more often in fawns under 1-year-old. The seropositivity of females was higher than in males.

**Conclusion:**

Wild ungulates can potentially represent a reservoir of new pathogenic livestock viruses. To study the prevalence and genetic diversity of wild ungulate herpesviruses, detailed molecular studies of the cervid herpesvirus 1, cervid herpesvirus 2, and elk herpesvirus 1 are necessary.

## Introduction

For a long time, the scientific community was interested in cattle diseases caused by viral pathogens in relation to agricultural production. However, in the past 15 years, there has been an increased interest in studying the spread of such pathogens in populations of wild artiodactyls. Researchers are trying to assess what role wild ungulates play in harboring and transmitting the infection. As more information becomes available, more species of wild animals are being identified as reservoirs of viral pathogens, expanding the range of identified hosts.

It has been shown that different viruses originally found in cattle, can be detected in different species of wild ungulates in the vast areas not only in North America, but also in Europe and Asia [1–4]. Researchers primarily study the seroprevalence of various wild ungulates to herpesviruses, which have a direct economic impact on cattle and sheep livestock [5–8]. The causative agent of infectious bovine rhinotracheitis is bovine alphaherpesvirus 1 (BoHV-1), one of the dangerous pathogens affecting cattle. It belongs to *Alphaherpesvirinae* subfamily. Primary infection signs might be accompanied by various clinical manifestations, such as rhinotracheitis or vulvovaginitis; the virus can induce abortions in cows, balanoposthitis in bulls, and systemic infections in calves during their 1^st^ months of life. The latest published scientific data confirm the presence of antibodies to BoHV-1 in various groups of wild artiodactyls. According to American researchers, in 2007, the seropositivity of Alaskan caribou (*Rangifer tarandus*) to BoHV-1 was 47% [9]. Studies conducted in 2011–2012 on yaks (*Bos mutus)* from different parts of the Qinghai-Tibet Plateau in China showed the presence of antibodies to BoHV-1 in 27.9–44.6% of animals [3]. In 2014, BoHV-1 was isolated from 5 out of 225 buffaloes (*Bubalus bubalis*) in northeastern Argentina [10]. Studies of 64 wild ruminants from 13 different regions in Iran, including 25 mouflons (*Ovis orientalis*), 22 wild goats (*Capra aegagrus*), 9 Indian gazelles (*Gazella bennettii*), and eight goitered gazelles (*Gazella subgutturosa*), showed the absence of BoHV-1 antibodies. In contrast, the genetic material of BoHV-1 virus was detected in 1.5% of all cases [8]. In Iran, BoHV-1 strains were isolated from nasal and vaginal swabs in 3 out of 16 buffalo (*B. bubalis*) [11]. A large-scale study of wild ungulates was carried out by various scientific groups in Europe. A study conducted in Poland [12] showed that more than 50% of the studied 1194 wild deer were seropositive to alphaherpesviruses. Antibodies were detected in red deer (*Cervus elaphus*) (25.6%), fallow deer (*Dama dama*) (23.1%), and roe deer (*Capreolus capreolus*) (1.7%). BoHV-1 DNA and antibodies have been reported in populations of Norwegian reindeer (*R. tarandus*) and red deer in Ireland, France, and Belgium [7, 13, 14]. Bovine herpesvirus type 5 (BoHV-5), which also belongs to *Alphaherpesvirinae* [15, 16], circulates in populations of domestic ungulates. Although the presence of the virus in Eastern Europe and the USA cannot be ruled out, BoHV-5 in cattle is more frequently detected in South America (Brazil and Argentina) [16]. At the same time, there is practically no data on the presence of the pathogen in wild animals. Molecular studies show that several viruses that are phylogenetically related to alphaherpesviruses are found in wild ungulates: cervid herpesvirus 1 (CvHV-1), which causes conjunctivitis in red deer, cervid herpesvirus 2 (CvHV-2), and elk herpesvirus 1 (ElkHV-1), which causes subclinical genital infections in reindeer (*R. tarandus*) and elk (*Cervus elaphus canadensis*), respectively. Interestingly, a serological cross-link was established between these viruses and BoHV-1 using ELISA [17, 18]. Representatives of the subfamily *Gammaherpesvirinae*, bovine herpesvirus type 4 (BoHV-4) and herpesvirus type 6 (BoHV-6), have been studied rarely. Since it was discovered in Hungary in 1963, BoHV-4 has been identified in cattle all over the world [19–22]. It was also shown that BoHV-4 can infect American bison (*Bison bison*), buffalos (*Syncerus caffer*), sheep (*Ovis aries*) and goats (*Capra hircus*) [20]. BoHV-6, also known as bovine lymphotropic herpesvirus (BLHV), was first identified in the United States in 1998 in lymphoma samples and bovine peripheral blood cells. It has been later found in cattle in Canada, South America, and New Zealand [23–25]. In 2021, Rosato *et al*. [26] showed that cattle animals from Switzerland, United Kingdom, Finland, Belgium, and Germany were carriers of BoHV-6 with an overall frequency of 32%, ranging from 22% to 42% in different countries. Based on the data obtained, the authors concluded that BoHV-6 is ubiquitous in healthy cattle. In 2013, BoHV-6 DNA was first detected in nasal swabs of a calf with clinical signs of respiratory pathology in the Moscow region [27]. Furthermore, molecular studies have shown a wide prevalence of BoHV-6 in the same area [28]. Other *Gammaherpesvirinae*, such as elk gammaherpesvirus (Elk-LHV) or fallow deer lymphotropic herpesvirus (LHV), has also been identified in wild ungulates. However, their association with the disease is still unknown. It is believed that they can affect animal’s immune system and cause lymphoproliferative diseases in other species [29, 30]. There is no information on the circulation of these viruses in wild cervids on the territory of Eastern Europe. In 2009, Australian scientists attempted to compare the BoHV-1 seroprevalence in wild animals with the intensity of livestock production in the same area [31]. Their results showed that BoHVs were present in wild ruminant populations all around the world, but whether wild ungulates can transmit the viral infection to livestock remained unresolved. There are currently no reports on the prevalence of BoHVs in the wild fauna of the Russian Federation.

The research aimed to study the occurrence of BoHVs among wild artiodactyls from different areas of the Moscow region in the Russian Federation.

## Materials and Methods

### Ethical approval

Ethical approval is not required for this type of study. This study used samples obtained from game animals.

### Study period and location

The study was conducted from November 2019 to May 2022. The samples were collected from eight districts of the Moscow region (Russia).

### Sample collection

**Figure-1:**
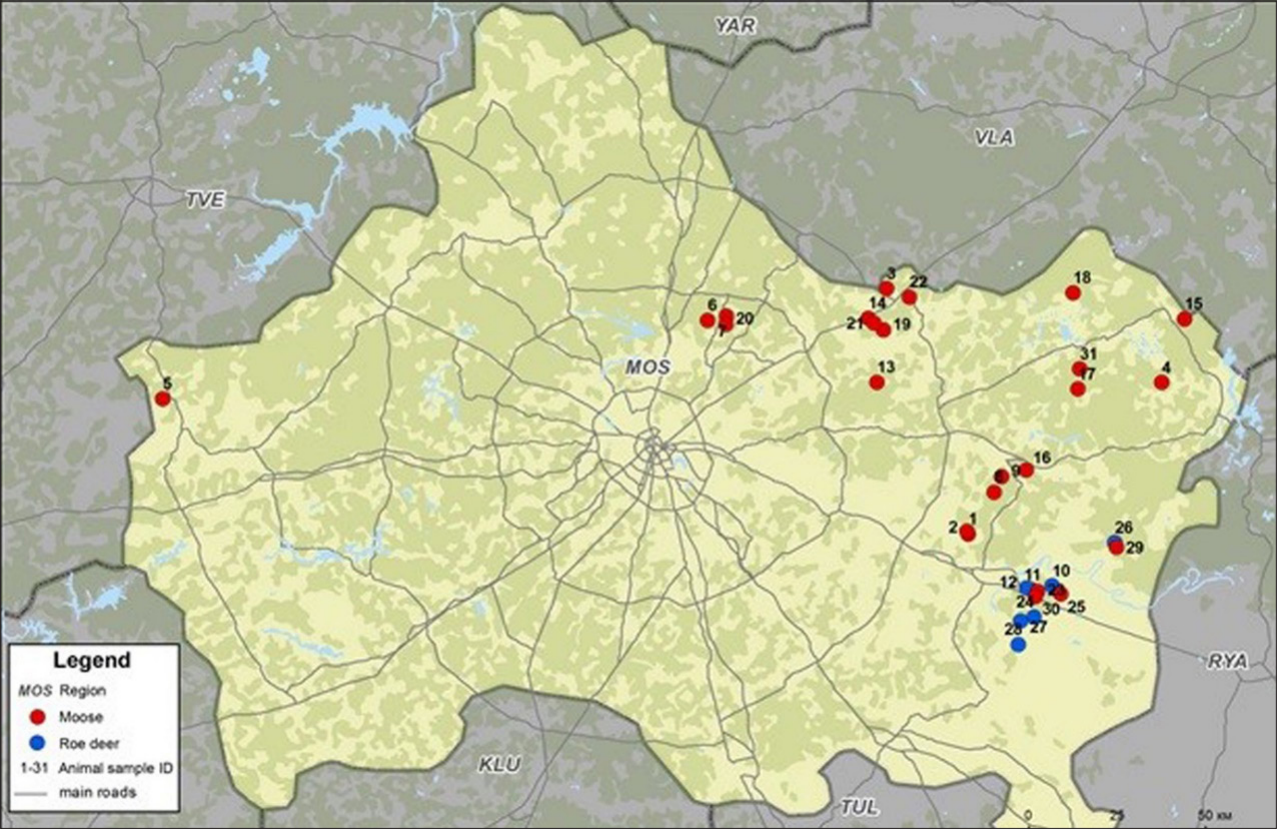
Localization of animal samples in the Moscow region [Source: Map was constructed usng QGIS-OSGeo-4W 3.18.2 program (https://qgis.org/ru/site/index.html)].

Samples used in the study were obtained from wild free-living artiodactyls, which were shot during the winter hunting season in 2019 in the Moscow region. Materials were collected from 31 animals, including 24 moose (*Alces alces*) and seven roe deer (Figure-1).

Animal descriptions and the place of their shooting are presented in Table-1.

**Table-1:** Animal descriptions.

Sample ID	Animal	Sex*	Age	Shooting locations
1M	Moose	F	>2	Kolomna district
2M	Moose	M	>2	Kolomna district
3M	Moose	F	>2	Orekhovo-Zuevo district
4M	Moose	F	>2	Shatura district
5M	Moose	F	5	Lotoshino district
6M	Moose	F	3	Schyolkovo district
7M	Moose	M	1.5	Schyolkovo district
8M	Moose	F	>2	Yegoryevsk district
9M	Moose	F	>2	Yegoryevsk district
10D	Roe deer	F	>2	Lukhovitsy district
11D	Roe deer	F	>2	Lukhovitsy district
12D	Roe deer	F	>2	Lukhovitsy district
13M	Moose	M	<1	Pavlovsky-Posad district
14M	Moose	F	4.5	Pavlovsky-Posad district
15M	Moose	M	3	Shatura district
16M	Moose	F	2.5	Yegoryevsk district
17M	Moose	M	1.5	Yegoryevsk district
18M	Moose	M	1.5	Shatura district
19M	Moose	M	<1	Orekhovo-Zuevo district
20M	Moose	F	>2	Schyolkovo district
21M	Moose	M	3.5	Orekhovo-Zuevo district
22M	Moose	F	<1	Orekhovo-Zuevo district
23M	Moose	M	1.5	Lukhovitsy district
24D	Roe deer	M	2	Lukhovitsy district
25M	Moose	M	1.5	Lukhovitsy district
26D	Roe deer	F	>2	Lukhovitsy district
27D	Roe deer	F	>2	Lukhovitsy district
28D	Roe deer	F	>2	Lukhovitsy district
29M	Moose	F	>2	Lukhovitsy district
30M	Moose	M	2	Lukhovitsy district
31M	Moose	F	<1	Shatura district

* F=Female, M=Male

For the polymerase chain reaction (PCR) testing, 101 animal tissue samples, including parts of the nasal septum, upper tracheal rings, lung, heart, liver, kidneys, testicles, and nasal swabs, were examined. After the collection, all samples were frozen at the temperature of −18°C before they were studied. For the serological studies, postmortem blood samples were taken from 19 animals by puncture of heart cavities. To obtain serum, tubes with blood were left at room temperature (23°C) for 30 min and then centrifuged at 800× *g* for 10 min.

### Serological methods

A virus neutralization test was conducted on serum samples of moose and roe deer with a constant amount of the virus BoHV-1 isolate Kuibyshev-2006 (2lg TCD_50_) from the Collection of Strain from the «Federal Scientific Center – All-Russian Research Institute of Experimental Veterinary Medicine – Russian Academy of Sciences», Moscow. Each serum sample was examined in six repetitions.

MDBK cell culture was used for the production of the virus. Cultivation was carried out in polystyrene mats with a growth area of 75 cm^2^ under a non-ventilated lid and under the temperature of 37°C. The growth nutrient medium, IglaMEM (PanEco, Russia) was supplemented with 7% bovine blood serum. Reseeding of the culture was carried out once a week in a ratio of 1:3. Infection of the cell culture was carried out by the conventional method after the formation of a complete monolayer. Cytopathic effect was monitored daily using a low magnification inverted microscope until the a physical detachment of most of the monolayer from the substrate was noticed. Calculation of the infectious titer of the virus was carried out according to the Reed and Muench method [32].

The virus neutralization reaction was carried out using a micromethod in 96-well culture plates with a constant dose of the virus (2lg TCD_50_) according to the generally accepted method. The results were recorded 72 h after the reaction was set up. The final antibody titer was calculated by the Spearman-Kärber method with a 95% confidence interval [33].

### DNA extraction and PCR

Tissue samples were homogenized in 10 mL 0.9% sodium chloride. The homogenate was centrifuged at 500× *g* for 5 min; the supernatant was taken and used for further studies. DNA was extracted from 100 μL of the suspension using the RIBO-prep kit (AmpliSens, Russia). The samples were tested for the presence of BoHV-4 DNA by conventional PCR with specific primers [34].

BoHV-6 DNA conventional PCR reaction was made with specially designed primers. First, all the BoHV-6 sequences were downloaded from the GenBank database (The National Center for Biotechnology Information [NCBI]). Then, the sequences were aligned using the Clustal W algorithm, integrated into the AlignX module of the VectorNTI Advanced 11.0 package (InforMax, Inc., USA). Selected primers flanked the DNA polymerase gene (*Dpol*) region of BoHV-6 isolate Pennsylvania 47 (NCBI Reference Sequence: NC_024303.1), which is about 550 bp long. The specificity of primers was studied using the Nucleotide BLAST.

Experimental confirmation of the specificity of the BoHV-6 primers was obtained using DNA of 32 strains of various microorganisms, herpesviruses, and bovine samples. PCR products of expected length from bovine semen and serum samples were studied and confirmed by Sanger sequencing. Afterward, positive samples were used as positive PCR controls.

Amplification was performed in a 25 μL reaction mixture containing 2.5× PCR-mix2 blue (AmpliSens), 10 mM of dNTPs, 0.6 μM of both forward and reverse primers (Table-2) [34]. The conventional PCR reaction was carried out on “Tercyk” Multi-block Thermocycler (DNA-technology, Russia) under the conditions illustrated in Table-1. The PCR amplicons were analyzed by running 10 μL of the PCR products on a 1.8% agarose gel stained with ethidium bromide (0.5 μg/mL) in comparison with a GeneRuler 100 bp DNA Ladder (Thermo Scientific™, Lithuania), visualized under the UV light, and photographed by Infinity 1500/36M Xpress Gel documentation system (Vilber Lourmat, France).

**Table-2:** List of oligonucleotides and PCR conditions for BoHV-4 and BoHV-6.

Virus	Target region	Primer name	Sequence 5’- 3’	Size (bp)	PCR condition	References
BoHV-4	*tk*	BoHV-4 F BHV-4 R	TTGATAGTGCGTTGTTGGGATGTGG CACTGCCCGGTGGGAAATAGCA	260	95°C - 5 min, 45 cycles (95°C - 10 s, 65°C - 20 s, 72°C- 20 s), 72°C- 5 min	[34]
BoHV-6	*Dpol*	BoHV-6-pol-F BoHV-6-pol-R	ACAGACGGGCAGCAGATAAG ATGGTTCGCCCCTGTAGAGT	554	95°C - 5 min, 42 cycles (95°C - 10 s, 55°C - 10 s, 72°C - 20 s), 72°C - 5 min	This study

PCR=Polymerase chain reaction, BoHV-4=Bovine herpesvirus type 4, BoHV-6=Bovine herpesvirus type 6

Detection of BoHV-1 DNA was based on the amplification of the *gE* gene fragment using the RINOKOR RT-PCR kit (AmpliSens) on a RotorGene Q (Qiagen, Germany).

## Results

### Sampling and animal distribution

Animals, 24 moose and seven roe deer, were hunted in 8 districts of the Moscow region. More female (n = 19) than male (n = 12) samples were studied. Animals were classified into three age categories based on their morphological characteristics, including body size, tooth wear, and antler growth: fawns (<1-year-old), yearlings (1–<2-years-old), and adults (≥2-years-old). Most of the animals were adults (n = 22), and 1-year-olds (n = 5) and fawns (n = 4) were approximately equal in quantity.

### Serological studies

The results of serological studies are presented in Table-3. Serum samples of 9/31 animals contained antibodies to the BoHV-1 with a titer from 0.67 to 3.75. Antibodies were found in serum samples of 3/5 roe deer from the Lukhovitsy district. Out of the 14 tested serum samples taken from moose, BoHV-1 antibodies were detected in six samples from 4 districts of the Moscow region. The maximum titer was detected in the serum sample of an adult moose shot in the Shatura district.

**Table-3:** Average titer of antibodies to BoHV-1 in tested wild animals.

Sample ID	Animal	Sex	Age group	Sampling sites	Mean antibody titer and 95% CI, log2
3M	Moose	F	Adult	Orekhovo-Zuevo district	0
4M	Moose	F	Adult	Shatura district	3.75 ± 0.10
6M	Moose	F	Adult	Schyolkovo district	0
7M	Moose	M	Yearling	Schyolkovo district	0
8M	Moose	F	Adult	Yegoryevsk district	0
9M	Moose	F	Adult	Yegoryevsk district	0
10D	Roe deer	F	Adult	Lukhovitsy district	2.00 ± 0.17
11D	Roe deer	F	Adult	Lukhovitsy district	2.17 ± 0.14
12D	Roe deer	F	Adult	Lukhovitsy district	2.67 ± 0.09
14M	Moose	F	Adult	Pavlovsky-Posad district	0
15M	Moose	M	Adult	Shatura district	0
16M	Moose	F	Adult	Yegoryevsk district	1.92 ± 0.12
17M	Moose	M	Yearling	Yegoryevsk district	0.67 ± 0.19
18M	Moose	M	Yearling	Shatura district	0
19M	Moose	M	Fawn	Orekhovo-Zuevo district	2.42 ± 0.11
20M	Moose	F	Adult	Schyolkovo district	2.00 ± 0.19
22M	Moose	F	Fawn	Orekhovo-Zuevo district	2.42 ± 0.12
24D	Roe deer	M	Adult	Lukhovitsy district	0
27D	Roe deer	F	Adult	Lukhovitsy district	0

F=Female, M=Male, BoHV-1=Bovine herpesvirus type 1

### PCR screening

DNA material of BoHV-4 and BoHV-6 was not found by conventional PCR in all 31 animals. At the same time, BoHV-1 DNA was detected using a real-time PCR in samples taken from 2 adult roe deer shot in the Lukhovitsy district of the Moscow region (sample ID 12D - Ct 31.04 and sample ID 27D - Ct 31.54).

## Discussion

Samples taken from one of the roe deer contained both BoHV-1 antibodies and viral DNA, which may indicate a prolonged animal contact with the virus. At the same time, in another roe deer BoHV-1 DNA was detected in a nasal swab sample and there were no antibodies in the serum, which may indicate the initial stage of infection. The presence of the BoHV-1 DNA calls attention on the ongoing infection in wild ruminants in the European part of Russia. This does not exclude the possibility that wild ruminants can serve as a reservoir of an infectious agent and, therefore, pose a threat of introducing infection onto the territory of livestock farms.

Since vaccination of wild artiodactyl animals against BoHV-1 is not carried out in Russia, positive results of serological studies may refer to the contact of these animals with the BoHV-1 virus.

**Figure-2:**
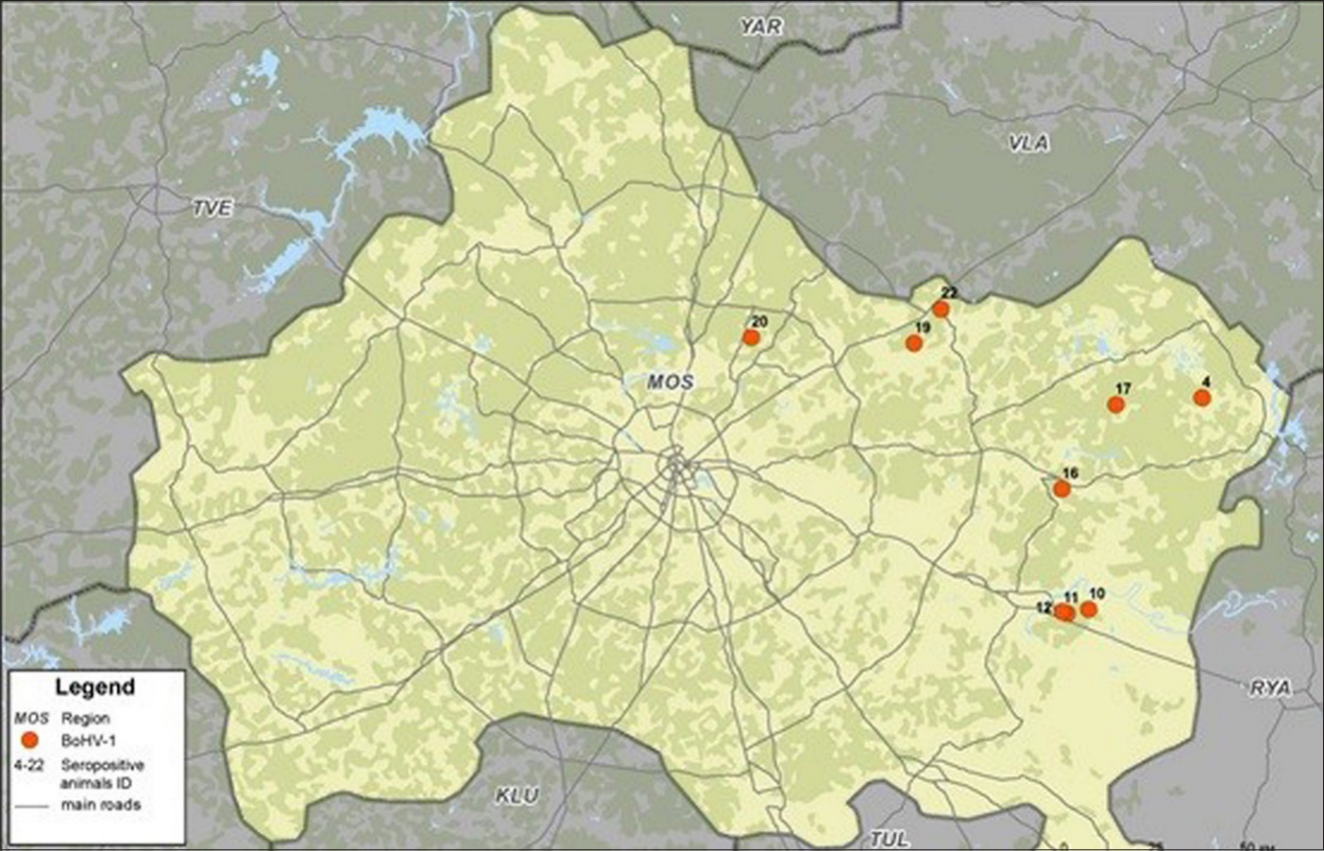
Localization of Bovine herpesvirus type 1 seropositive animals in the Moscow region [Source: Map was constructed using QGIS-OSGeo-4W 3.18.2 program (https://qgis.org/ru/site/index.html)].

All 19 samples for serological studies were colleccted from districts of the Eastern and Southeastern regions of Moscow (Figure-1). Antibodies to BoHV-1 were discovered in half of these samples (Figure-2). It is impossible to exclude viral circulation in other areas of the Moscow region, where a relatively high density of wild ruminants is observed.

**Figure-3:**
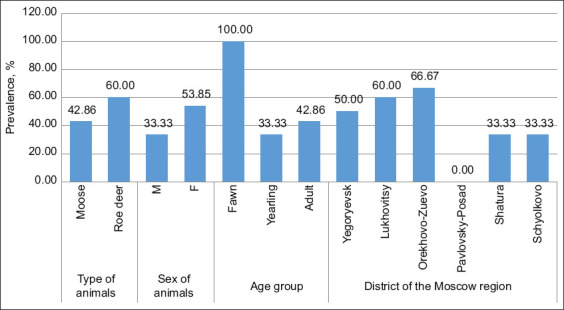
Analysis of the seropositivity by animal species, sex, age, and district.

Figure-3 shows the results of seroprevalence tests in different groups. Antibodies to BoHV-1 were found in 60% of the tested roe deer and approximately 43% of the tested moose serum samples. Regarding age-related seroprevalence, it can be concluded that most of the positive serum samples were detected in fawns, then in adult animals, and the least amount was found in 1-year-old animals. Differences in seropositivity of various groups of animals can be explained by the social structure of cervid groups during wintertime. In winter, roe deer form family groups of 10–15 individuals that consist of females with fawns. In moose, social structure is similar, but the groups are comprised of 3–4 individuals. In such communities, the transmission of the virus may occur more frequently between females. Yearlings join such groups less often, and fawns, born in summer may still have colostral antibodies. In our study, the seropositivity of the tested female serum samples is higher than those of males, which is also explained by the peculiarity of animal behavior – adult males often live separately.

Our results generally correlate with the data on BoHV-1 seropositivity in wild artiodactyls in other countries. Thus, in yaks living in the Tibetan Plateau in China, level of BoHV-1 antibodies varied from 27.9% to 44.6%, depending on the animal habitat [3]. In the study by Lillehaug *et al*. [13], more than 3000 Norway cervids serum samples were tested. Antibodies to BoHV-1 were detected in 3% of roe deer samples, but all moose samples were negative. Overall, the value of seropositivity was 28.5%. In a large-scale study of alphaherpesviruses (BoHV-1 and CvHV-1) in free-living ruminants in Poland, seroprevalence ranged from 0% to 100% in different areas [12]. Due to the small number of analyzed samples, our results do not allow us to determine the general seropositivity of Moscow region cervids to alphaherpesviruses. However, it is now known that wild artiodactyls that live near Moscow had contact with BoHV-1, and this statement has a correlation with the global trend.

In addition, our study has confirmed the results of the study by Shuliak *et al*. [35], who demonstrated the presence of infectious bovine rhinotracheitis virus type 1 circulation in wild artiodactyls in Russia. Data from European scientists also validated the fact that BoHV-1 herpesvirus spread among wild artiodactyls in the countries of Eastern Europe, especially Poland, where the virus was noted in fallow deer, red deer, and, to a lesser extent, roe deer [12]. However, in our study, cross-serological reactions with related BoHV-1 herpesviruses in deer (CvHV-1, CvHV-2, and ElkHV-1) cannot be ruled out. For the evaluation of the circulation of these viruses additional PCR studies are required.

## Conclusion

PCR and serological methods showed that the studied moose and roe deer were in contact with *Alphaherpesvirinae* BoHV-1, but *Gammaherpesvirinae* BoHV-4 and DNA were not detected in any of the samples. Further studies on the occurrence of other wild deer herpesviruses are needed to exclude cross-serological reactions with BoHV-1, as well as the studies of a larger number of animals from other districts of the Moscow region.

## Authors’ Contributions

SY and AVP: Designed the study and drafted the manuscript. AVP: Collected samples. SY and MSK: Conducted PCR testing. AVP and ERS: Performed the serological work. SY: Revised the manuscript. All authors have read and approved the final manuscript.

## References

[1] Casaubon J, Vogt H.R, Stalder H, Hug C, Ryser-Degiorgis M.P (2012). Bovine viral diarrhea virus in free-ranging wild ruminants in Switzerland:Low prevalence of infection despite regular interactions with domestic livestock. BMC Vet. Res.

[2] Conner M.M, Ebinger M.R, Blanchong J.A, Cross P.C (2008). Infectious disease in cervids of North America:Data, models, and management challenges. Ann. N. Y. Acad. Sci.

[3] Han Z, Gao J, Li K, Shahzad M, Nabi F, Zhang D, Li J, Liu Z (2016). Prevalence of circulating antibodies to bovine herpesvirus 1 in Yaks (*Bos grunniens*) on the Qinghai-Tibetan Plateau, China. J. Wildl. Dis.

[4] Kalman D, Egyed L (2005). PCR detection of bovine herpesviruses from nonbovine ruminants in Hungary. J. Wildl. Dis.

[5] Fabisiak M, Sałamaszynska A, Stadejek T (2018). Detection of seroconversion to bovine herpesvirus 1 related alphaherpesvirus and bovine viral diarrhea virusin Polish free-living deer. Pol. J. Vet. Sci.

[6] Frolich K, Hamblin C, Parida S, Tuppurainen E, Schettler E (2006). Serological survey for potential disease agents of free-ranging cervids in six selected national parks from Germany. J. Wildl. Dis.

[7] Graham D.A, Gallagher C, Carden R.F, Lozano J.M, Moriarty J, O'Neill R (2017). A survey of free-ranging deer in Ireland for serological evidence of exposure to bovine viral diarrhoea virus, bovine herpes virus-1, bluetongue virus and Schmallenberg virus. Irel. Vet. J.

[8] Hemmatzadeh F, Boardman W, Alinejad A, Hematzade A, Moghadam M.K (2016). Molecular and serological survey of selected viruses in free-ranging wild ruminants in Iran. PLoS One.

[9] Evans A.L, das Neves C.G, Finstad G.F, Beckmen K.B, Skjerve E, Nymo I.H, Tryland M (2012). Evidence of alphaherpesvirus infections in Alaskan caribou and reindeer. BMC Vet. Res.

[10] Maidana S.S, Konrad J.L, Craig M.I, Zabal O, Mauroy A, Thiry E, Crudeli G, Romera S.A (2014). First report of isolation and molecular characterization of bubaline herpesvirus 1 (BuHV1) from Argentinean water buffaloes. Arch Virol.

[11] Hedayat N, Hajikolaei M.R.H, Seyfi Abad Shapouri M.R (2020). Isolation and identification of bubaline herpesvirus 1 (BuHV-1) from latently infected water buffalo (*Bubalus bubalis*) from Iran. Trop. Anim. Health Prod.

[12] Rola J, Larska M, Socha W, Rola J.G, Materniak M, Urban-Chmiel R, Thiry E, Żmudziński J.F (2017). Seroprevalence of bovine herpesvirus 1 related alphaherpesvirus infections in free-living and captive cervids in Poland. Vet. Microbiol.

[13] Lillehaug A, Vikøren T, Larsen I.L, Akerstedt J, Tharaldsen J, Handeland K (2003). Antibodies to ruminant alpha-herpesviruses and pestiviruses in Norwegian cervids. J. Wildl. Dis.

[14] Thiry E, Vercouter M, Dubuisson J, Barrat J, Sepulchre C, Gerardy C, Meersschaert C, Collin B, Blancou J, Pastoret P.P (1988). Serological survey of herpesvirus infections in wild ruminants of France and Belgium. J. Wildl. Dis.

[15] Bartha A, Hajdu G, Aldasy P, Paczolay G (1969). Occurrence of encephalitis caused by infectious bovine rhinotracheitis virus in calves in Hungary. Acta Vet. Acad. Sci. Hung.

[16] Del Medico Zajac M.P, Ladelfa M.F, Kotsias F, Muylkens B, Thiry J, Thiry E, Romera S.A (2010). Biology of bovine herpesvirus 5. Vet. J.

[17] Thiry J, Widén F, Grégoire F (2007). Isolation and characterisation of a ruminant alphaherpesvirus closely related to bovine herpesvirus 1 in a free-ranging red deer. BMC Vet. Res.

[18] Azab W, Dayaram A, Greenwood A.D, Osterrieder N (2018). How host specific are herpesviruses?Lessons from herpesviruses infecting wild and endangered mammals. Annu. Rev. Virol.

[19] Cobb S.P, Banks M, Russell C, Thorne M (2006). Bovine lymphotropic herpesvirus in a UK dairy herd. Vet. Rec.

[20] Mishchenko V.A, Mishchenko A.V, Dumova V.V, Shevchenko A.A, Chernykh O.Y (2013). Vet. Kubany.

[21] Nefedchenko A.V, Koteneva S.V, Glotova T.I, Glotov A.G, Yuzhakov A.G, Zaberezhny A.D (2019). Detection of bovine herpesvirus 4 DNA in cattle by real-time PCR. Vopr Virusol.

[22] Williams L.B.A, Fry L.M, Herndon D.R, Franceschi V, Schneider D.A, Donofrio G, Knowles D.P (2019). A recombinant bovine herpesvirus-4 vectored vaccine delivered via intranasal nebulization elicits viral neutralizing antibody titers in cattle. PLoS One.

[23] Gagnon C.A, Allam O, Drolet R, Tremblay D (2010). Quebec:Detection of bovine lymphotropic herpesvirus DNA in tissues of an aborted bovine fetus. Can Vet. J.

[24] de Oliveira C.H, de Oliveira F.G, Gasparini M.R, Galinari G.C, Lima G.K, Fonseca A.A, Barbosa J.D, Barbosa-Stancioli E.F, Leite R.C, Dos Reis J.K (2015). Bovine herpesvirus 6 in buffaloes (*Bubalus bulalis*) from the Amazon region, Brazil. Trop Anim Health Prod.

[25] de Boer M.W, Zheng T, Buddle B.M, McDougall S (2014). Detection of bovine herpesvirus type 4 antibodies and bovine lymphotropic herpesvirus in New Zealand dairy cows. N Z Vet J.

[26] Rosato G, Subira A.R, Al-Saadi M, Michalopoulou E, Verin R, Dettwiler M, Nordgren H, Chiers K, Grobmann E, Kohler K, Suntz M, Stewart J.P, Kipar A (2021). Gammaherpesvirus infections in cattle in Europe. Viruses.

[27] Yurov K.P, Alekseenkova S.V, Pchelnikov A.V (2013). Identification of Bovine Lymphotropic Gammaherpesvirus in the Nasal Secretions of Sick Calves. Theory and Practice of Actual Research:Proceedings of the 5^th^ International Scientific and Practical Conference, Krasnodar, September 17, 2013.

[28] Pchelnikov A.V, Yatsentyuk S.P (2021). Detection of the genetic material of gammaherpesviruses in livestock farms of the Moscow and Tver regions. J. Vet.

[29] Auer A, Schweitzer L, Kübber-Heiss A, Posautz A, Dimmel K, Seitz K, Beiglböck C, Riedel C, Rümenapf T (2022). Porcine circoviruses and herpesviruses are prevalent in an Austrian game population. Pathogens.

[30] Zhu H, Liu H, Yu X, Zhang J, Jiang L, Chen G, Feng Z, Li Y, Feng T, Zhang X (2018). Evidence of two genetically different lymphotropic herpesviruses present among red deer, sambar, and milu herds in China. J. Vet. Sci.

[31] Cripps J.K, Pacioni C, Scroggie M.P, Woolnough A.P, Ramsey D.S.L (2019). Introduced deer and their potential role in disease transmission to livestock in Australia. Mamm. Rev.

[32] Reed L.J, Muench H (1938). A simple method of estimating fifty percent endpoints. Am. J. Epidemiol.

[33] Thrusfield M, Thrusfield M (1986). Serological epidemiology. Veterinary Epidemiology.

[34] Bayoumi Y, Sobhy N, Morsi A, El-Neshwey W, El-Seddawy N, Abdallah A (2021). Clinical and histopathological studies on neurodegeneration and dysautonomia in buffalo calves during foot-and-mouth disease outbreaks in Egypt. Vet. World.

[35] Shuliak A.F, Gorbatov A.V, Stafford V.V, Loschinin M.N, Velichko G.N, Sokolova N.A, Zhuravleva E.A, Ishkova T.A, Nosova M.V (2020). Mixed infection among wild ruminants on hunting grounds. J. Vet.

